# Mining of potential microRNAs with clinical correlation - regulation of syndecan-1 expression by miR-122-5p altered mobility of breast cancer cells and possible correlation with liver injury

**DOI:** 10.18632/oncotarget.25589

**Published:** 2018-06-15

**Authors:** YihHuei Uen, Jin-Wun Wang, ChiaChen Wang, Yaoyun Jhang, Jo-Yun Chung, TingTing Tseng, MingJen Sheu, ShaoChen Lee

**Affiliations:** ^1^ Department of Surgery, ChiMei Hospital, Chiali, Jiali Dist., Tainan City, Taiwan; ^2^ Department of Surgery, Asia University Hospital, Taichung City, Taiwan; ^3^ Department of Biotechnology, Asia University, Taichung City, Taiwan; ^4^ Department of Surgery, ChiMei Hospital, Chiali, Jiali Dist., Tainan City, Taiwan; ^5^ School of Medicine, Fu Jen Catholic University, Xinzhuang Dist., New Taipei City, Taiwan; ^6^ Department of Dermatology, Cardinal Tien Hospital, Xindian Dist., New Taipei City, Taiwan; ^7^ Department of Hepato-Gastroenterology, ChiMei Hospital, Yongkang Dist., Tainan City, Taiwan

**Keywords:** microRNA, syndecan-1, breast cancer, exosome

## Abstract

MicroRNAs are small noncoding RNAs acting as novel biomarkers of various diseases and potential regulators of protein expression and functions. Syndecan-1 is the heparan sulfate proteoglycan associated with malignancy of various cancers, including breast cancer. In this study, we proposed a experimental workflow to investigate potential microRNAs that regulate SDC1 expression and affect breast cancer cell mobility.

MicroRNA candidates were selected from available Gene Expression Omnibus datasets on breast malignancy. Further *in silico* duplex hybridization and multiplex PCR approach were used to screen potential microRNAs. Analysis showed increased syndecan-1 expression but decreased miR-122-5p level upon breast malignancy. Western blot and *in vitro* luciferase assay confirmed the targeting of 3′-untranslated region of syndecan-1 and suppression of syndecan-1 expression by miR-122-5p. The suppression of syndecan-1 expression by miR-122-5p or shRNAs against syndecan-1 increased breast cancer cell mobility; while overexpression of syndecan-1 inhibited cell mobility. In further, miR-122-5p was enriched in liver cell-derived exosomes that was able to suppress syndecan-1 expression and increase cell mobility in breast cancer cells.

In conclusion, our results suggested the downregulation of SDC1 by miR-122-5p or liver-cell-derived exosomes would enhance breast cancer cell mobility. Metastasis or mobility of breast cancer cells might be affected by circulating miR-122-5p and not directly correlated with progression of breast cancer.

## INTRODUCTION

Syndecan-1 (SDC1) is a heparan sulfate proteoglycan that acts as a binding acceptor for many soluble growth factors, cytokines, chemokines, and membrane-bound receptors [1]. SDC-1 regulates cellular activity through cell signaling associated with cell proliferation, differentiation, and cell–matrix interaction [2–4]. Increased levels of SDC1 are associated with the malignancy of various cancers [5], including breast cancer [6, 7]. SDC1 expression was decreased or increased in various tumor types and associated with histologic grades or malignancy. Higher SDC1 expression was associated with higher histologic grade and inversely related to hormonal receptor status of breast cancer cells [8]. Several literatures implied that distant metastasis of breast cancer would correlate with acute liver failure [9, 10]. However, no possible mechanism was proposed to explain the liver-specific metastasis.

MicroRNAs (miRNAs) are small ribonucleotides with 18~24 residues that regulate the expression of target proteins [11]. In general, miRNAs target the 3ʹ-untranslated region (3ʹUTR) of target mRNAs and result in translational repression or mRNA degradation [12, 13]. Thus, miRNAs are key regulators of cell proliferation, apoptosis, tumor formation, and tumor metastasis [14]. Recently, circulating miRNAs had been found in the microvesicles (or exosomes) present in most biofluids, including serum. The miRNAs might act as ectopic stimuli and communicators between donor and target cells and alter gene expression [15].

In this study, we analyzed available Gene Expression Omnibus (GEO) data from patients with breast cancer to search potential miRNAs regulating SDC1 expression. The role of miR-122-5p in suppressing SDC1 expression was validated. The presence of exosomal miRNA-122-5p secreted from liver cells would influence breast cancer mobility through SDC1 downregulation, that a mechanism affecting the metastasis of breast cancer was proposed.

## RESULTS

### Strategy to screen potential miRNAs in SDC1 regulation

**Figure 1:**
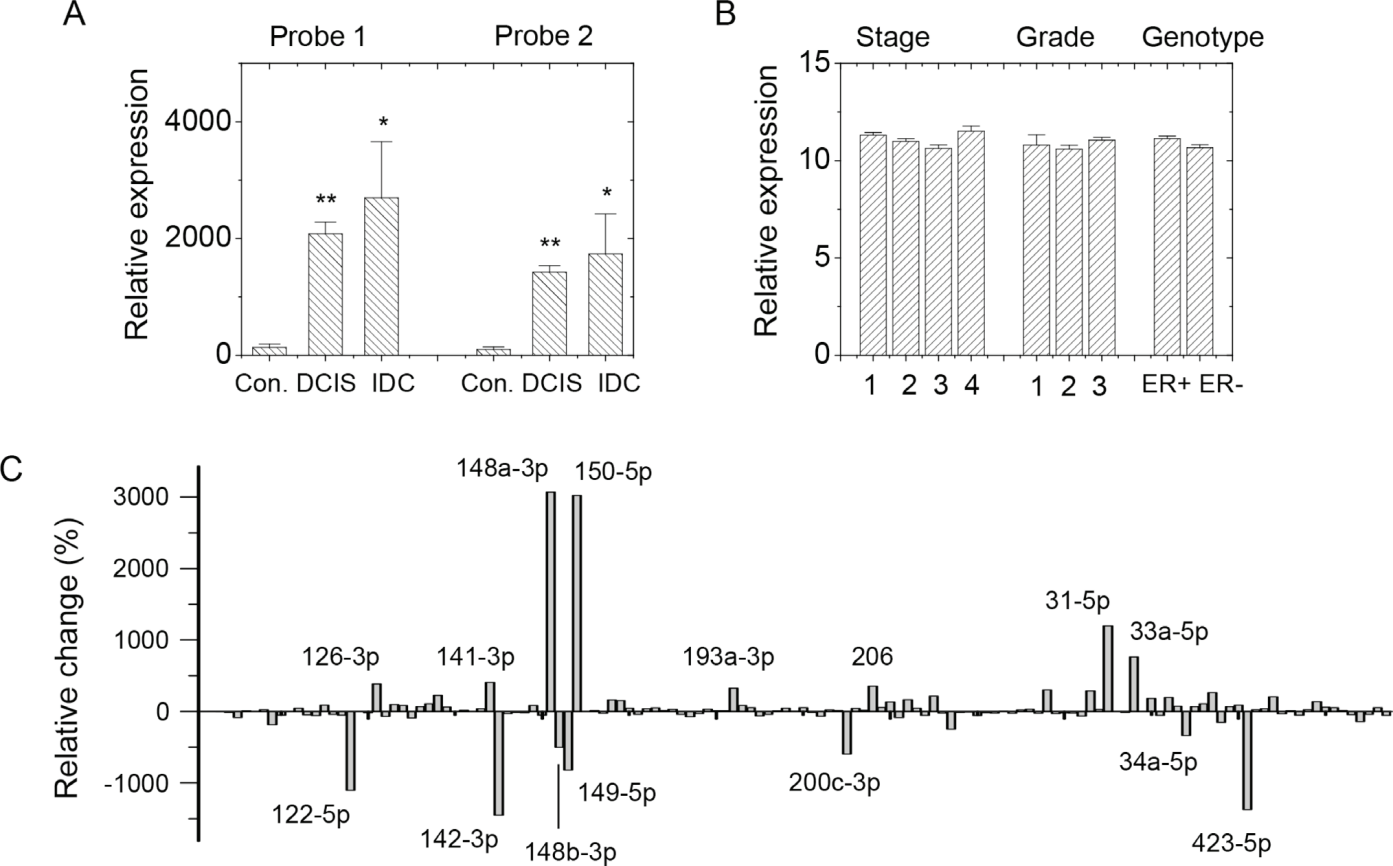
SDC1 and miRNA expression in GEO profile (**A**) SDC1 expression profile in GDS3853 showed increased expression levels in ductal carcinoma *in situ* (DCIS; *n* = 9) and invasive ductal carcinoma (IDC; *n* = 5) comparing with healthy breast tissue (*n* = 5). Two nucleotide probes against SDC1 were used. (**B**) SDC1 expression in datasets GDS4056 and GDS4057 showed no significant difference in breast cancer patients at different pre-chemotherapy stages (*n* = 2, 40, 80, respectively), BMN grades (*n* = 2, 38, 74, respectively), or ER+/ER– genotypes (*n* = 84 and 58, respectively). (**C**) Comparison in relative change of miRNA expressions in normal tissues (*n* = 5) and malignant tissues (*n* = 93) using GEO data GSE7842. Data were mean ± S.E. ^**^p < 0.01. ^*^p < 0.05.

SDC1 was demonstrated to be highly expressed in patients with malignant breast cancer, which was correlated with poor prognoses and aggressive phenotypes [6, 16]. A strategy to screen potential miRNAs regulating SDC1 expression in breast cancer cells was established. First, we analyzed GEO data for SDC1 expression and miRNA profiling of clinical samples from breast cancer patients. SDC1 expression profile in GDS3853 showed increased expression levels in ductal carcinoma *in situ* (DCIS) and invasive ductal carcinoma (IDC) compared with those in healthy breast tissue (Figure 1A). SDC1 expression did not change significantly in patients with breast cancer at different prechemotherapy stages, different BMN grades, or estrogen receptor (ER) ER+/ER– genotypes as revealed in GEO profiles GDS4056 and GDS4057 (Figure 1B). Those suggested the involvement of SDC1 in initial stages of transformation and contribution to the malignancy of breast cancer cells.

**Figure 2:**
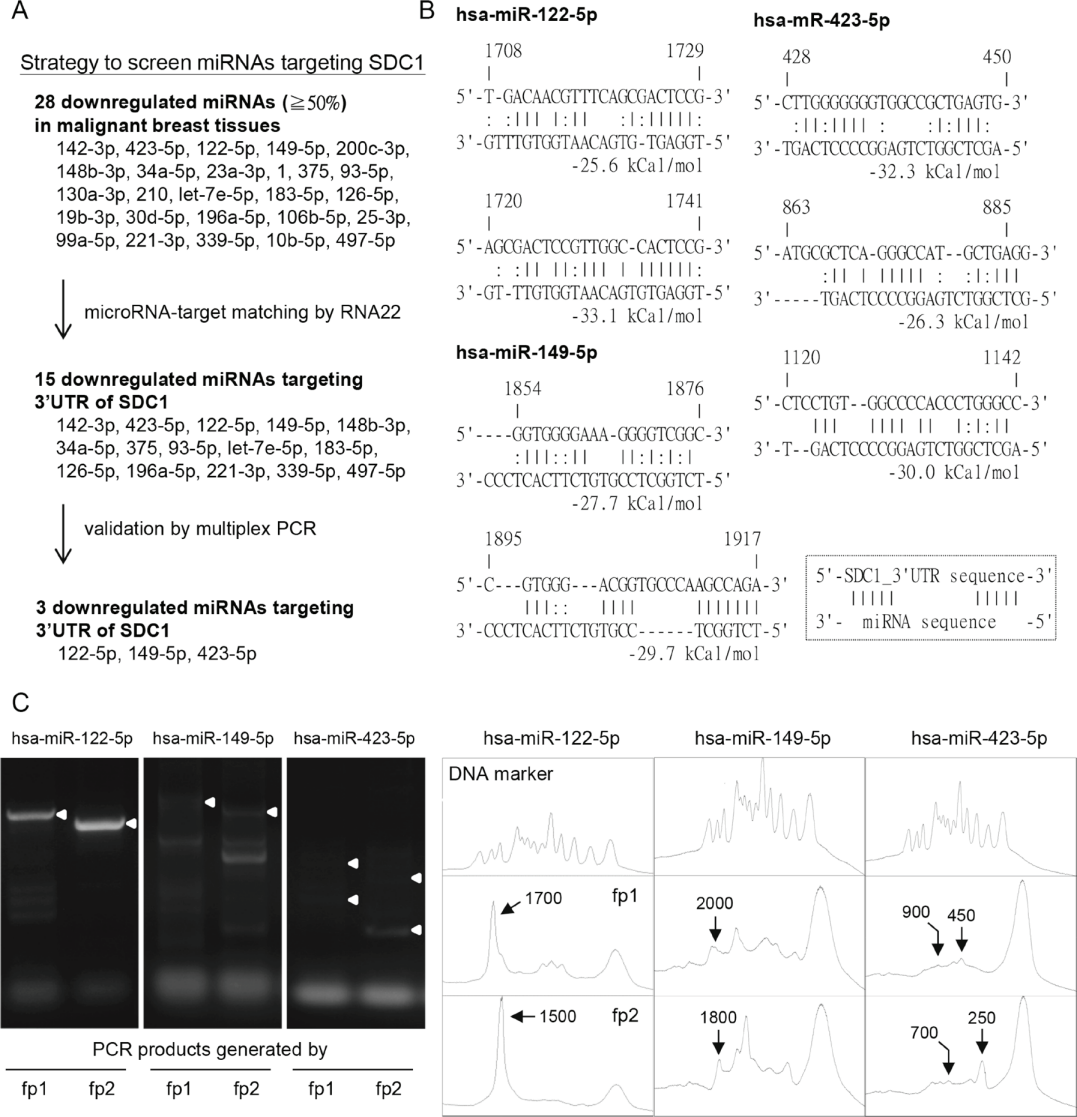
Potential microRNAs regulated SDC1 gene expression in breast cancer cells (**A**) Strategy to screen potential miRNAs regulating SDC1 protein expression. The downregulated miRNAs in GSE7842 were listed and filtered by RNA22 tools and multiplex PCR approach. Three downregulated miRNAs (hsa-miR-122-5p, -149-5p, and -423-5p) were selected. (**B**) Prediction of miRNA-SDC1_3′UTR duplex by RNA22 algorithm with folding energy of heteroduplex ≥ 25 kcal/mol (**C**) Multiplex PCR approach was used to validate the microRNA targeting sites. Two site-specific forward primers (fp1 and fp2), which targeted at locations with defined interval, were used individually in two independent PCR reactions. The histograms were generated by ImageJ to allocate the sizes of PCR products. The PCR products generated by site-specific forward primers (fp1 or fp2), which were consistent with prediction in Figure 2B were indicated by white arrowheads in the gels and arrows in the histograms. The peaks of DNA marker corresponded to the DNA size in 100, 200, 300, 400, 500, 600, 700, 800, 900, 1000, 1500, 2000, and 3000 bp, respectively.

Second, we screened potential downregulated miRNAs correlating with SDC1 upregulation. GEO profiling of miRNA expression levels between normal tissue and breast carcinoma (GSE7842) suggested several miRNAs acted as tumor markers [17]. GEO data collected clinical samples of normal breast tissues and tumor tissues at different tumor stages and with different BMN grades, vascular invasion indexes, prognosis indexes, and ER expression levels. Comparison of averaged data from normal tissues and tumor tissues showed relative changes in miRNA expression levels (Figure 1C). Totally, 28 miRNAs (Figure 2A) were characterized as downregulated miRNAs in malignant breast tissues.

Third, bioinformatics analysis was further used to screen potential miRNAs regulating SDC1 expression (Figure 2A). RNA22 would predict the targeting sites on the 3ʹUTR of SDC1 by these candidate miRNAs. RNA22 adapted a pattern-based algorithm to determine miRNA-targeting sites on a user-defined nucleotide sequence without cross-organism conservation constraints [18]. Totally, 15 potential miRNAs were found with folding energy of miRNA-target heteroduplex less than −25 kcal/mol as determined by Vienna RNA package [19] (Figure 2A and 2B).

Forth, a novel multiplex PCR analysis was used to screen potential miRNAs in further. This *in vitro* assay was used to visualize the miRNA-targeting site experimentally [20]. The pcDNA3.1 plasmid with the 3ʹUTR sequence of *SDC1* gene was constructed and used as PCR template. Mature miRNA oligonucleotide was used as reverse primer and site-specific oligonucleotides targeting at upstreams of *SDC1*-3ʹUTR sequence were used as forward primers in the multiplex PCR [20]. Successful PCR products were generated only by higher affinity interaction existed between the miRNA and target sequences. To distinguish specific binding from nonspecific binding, two different forward primers (fp1 and fp2) that target at upstream sequences of *SDC1*-3ʹUTR with a 200-bp interval were designed (See Supplementary Table 1). Two individual PCR reactions were performed using reverse primer (mature miRNA) and site-specific forward primer (fp1 or fp2), and PCR products were displayed by agarose gels. As shown in Figure 2C, the targeting of miR-122-5p (as the reverse primer) at position 1,700 of the 3ʹUTR sequence along with the fp1 forward primer generated a 1,700-bp PCR product, while the PCR product generated by miR-122-5p and the fp2 forward primer generated a 1,500-bp fragment. The size of the PCR product was characterized by comparing with DNA markers in the histogram (Figure 2C). Only the miRNA-targeting that generated PCR products differing in size of 200-bp were considered as valid targeting. Of all the 15 candidate miRNAs, only hsa-miR-122-5p, -149-5p, and -423-5p were valid in the multiplex PCR, and the targeting sites were consistent with the RNA22 prediction (Figure 2B). The hsa-miR-122-5p targeted position 1,720 to 1,741 of the *SDC1*-3ʹUTR with higher folding energy (−33.1 kJ/mol) and with 7 base pairings at the seed region of miR-122-5p (Figure 2B). In addition, GEO data (GSE7842) showed decreased expression of miR-122-5p in patients with all stages and grades of breast cancer (Supplementary Figure 1A and 1B), which was correlated with aforementioned results of SDC1 expression. A dramatic decrease was observed in patients with prechemotherapy stage 1 or BMN grade 1 cancer, which implied the involvement of miR-122-5p in the early stages of breast cancer malignancy. However, the change for miR-149-5p and miR-423-5p were subtle comparing to that of miR-122-5p (Supplementary Figure 1A and 1B). We thus selected miR-122-5p to study in further.

### Regulation of SDC1 protein expression by hsa-miR-122-5p

The expression of SDC1 protein and hsa-miR-122-5p were examined in two breast cancer cell lines, MCF-7 and MDA-MB-231, with western blotting and PCR. MDA-MB-231 breast cancer cells were more mobile than MCF-7 cells. It was also reported that lower SDC1 expression was correlated with higher cell mobility of breast cancer cells [21]. Cell lysate were pretreated with heparinase to remove glycosaminoglycan chains, and SDC1 protein was visualized at 67-kDa by western blot. We demonstrated that MCF-7 cells expressed more SDC1 protein compared with that in MDA-MB-231 cells (Supplementary Figure 1C), which was consistent with previous publication [22]. It was also correlated with less mobility and invasiveness of MCF-7 cells. As shown in Supplementary Figure 1D, MCF-7 cells exhibited less miR-122-5p expression compared with MDA-MB-231 cells. These results suggested miR-122-5p level might correlated with SDC1 protein expression and breast cancer cell mobility.

**Figure 3:**
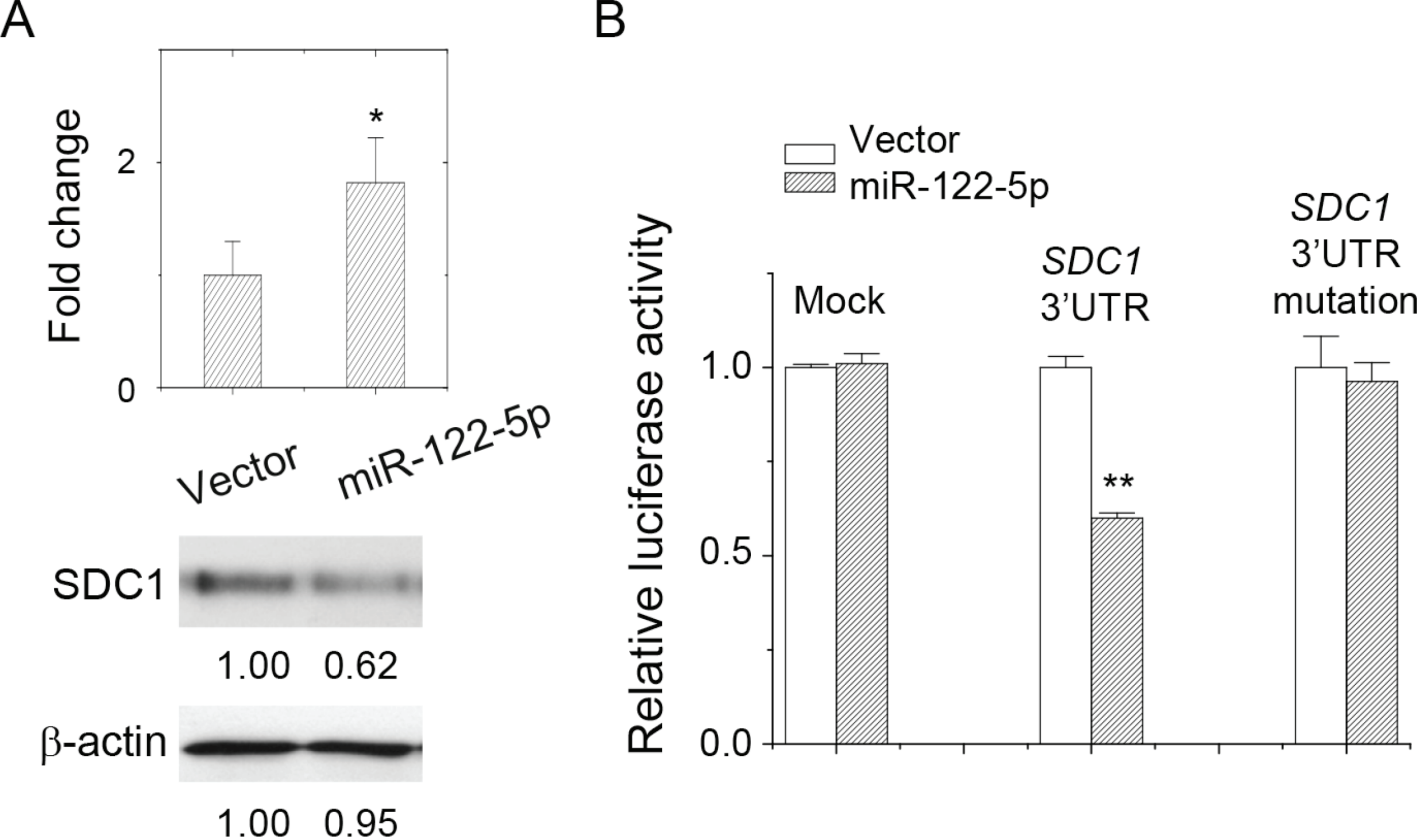
miR-122-5p targeted at SDC1_3′UTR and downregulated SDC1 expression (**A**) Overexpression of miR-122-5p inhibited SDC1 protein expression. (**B**) Luciferase reporter assay showed direct targeting of miR-122-5p at SDC1_3′UTR. Mutation at miRNA-binding sites at 3′UTR abolished inhibitory effect. Data were mean ± S.E. ^**^p < 0.01, ^*^p < 0.05.

To characterize whether SDC1 protein expression was downregulated by miR-122-5p, we constructed a miRNA expression plasmid that contained pre-miRNA-122 sequence. Hairpin RNA structure of miR-122 was expressed and further processed into mature miRNA-122-5p in mammalian cells. Upon transfection into MCF-7 cells, expression levels of miR-122-5p were quantified by PCR (Figure 3A). SDC1 mRNA expression was slightly reduced (data not shown), but protein expression was significantly inhibited by miR-122-5p transfection in MCF-7 cells (Figure 3A). This indicated that miR-122-5p might reduce SDC1 protein levels through translation blockage without significant mRNA degradation [13].

To confirm the direct targeting of miR-122-5p to *SDC1*-3ʹUTR, a luciferase reporter assay was performed. We cloned the 3ʹUTR of *SDC1* gene and ligated it into a luciferase reporter plasmid, which was used to validate miRNA–target interaction *in vitro*. As shown in Figure 3B, miR-122-5p suppressed luciferase activity only in the presence of SDC1 3ʹUTR. In addition, we mutated the 3ʹUTR nucleotide sequence at seed region of miRNA-targeting site (Supplementary Figure 1E), which was expected to reduce the affinity of miR-122-5p toward this specified targeting site. The 3ʹUTR mutation did eliminate the suppressive effect of miR-122-5p in luciferase reporter assay as well to abolish the PCR product in multiplex PCR (data not shown). These results confirmed the direct targeting and binding of miR-122-5p to *SDC1*-3ʹUTR.

### Downregulation of SDC1 protein by miR-122-5p enhanced cell mobility

**Figure 4:**
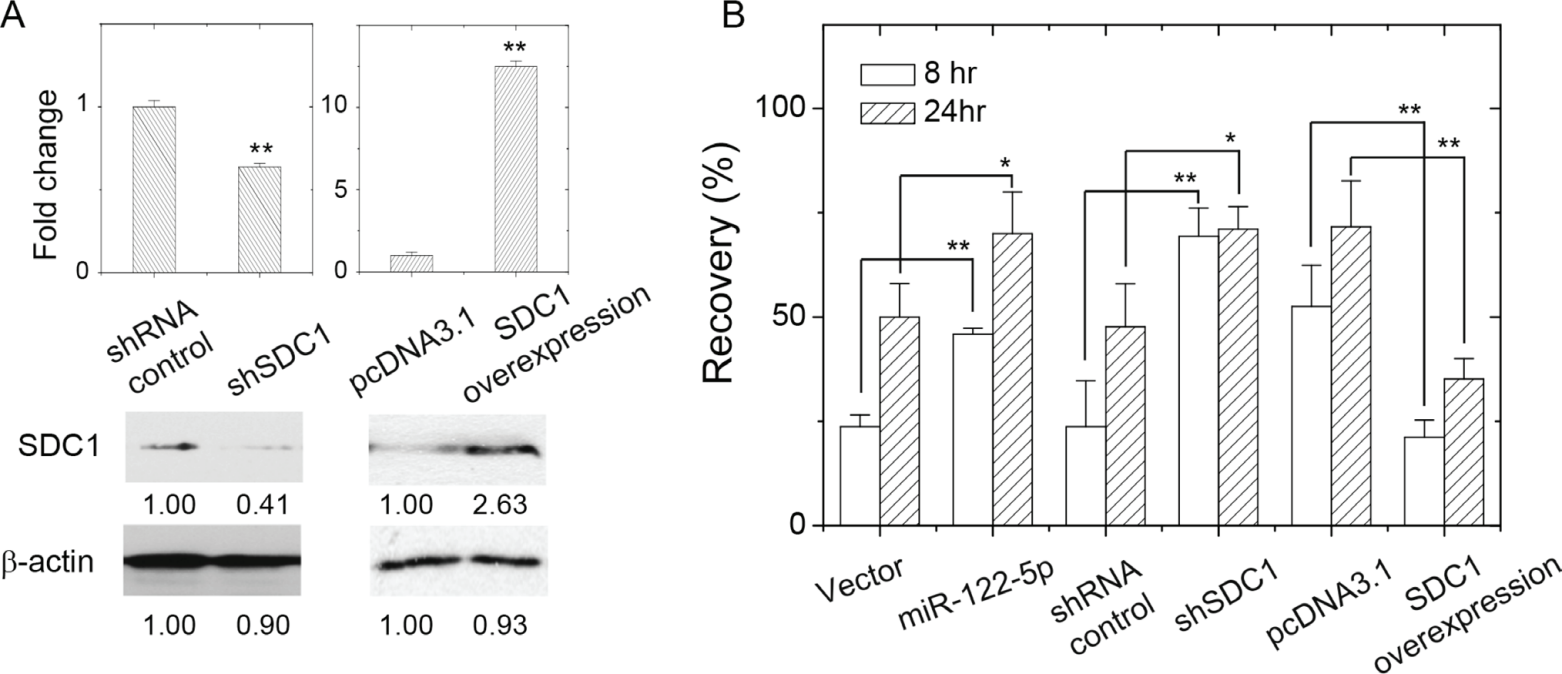
SDC1 expression level affected breast cancer cell mobility (**A**) Change of SDC1 protein expression by transfection of shRNA against SDC1 or SDC1 overexpression. (**B**) Cell mobility of MCF-7 cells as assayed by wound healing assay showed increased recovery by suppression of SDC1 expression (transfection of shRNA against SDC1 or miR-122-5p), and decreased mobility by SDC1 overexpression. Data were mean ± S.E. ^**^p < 0.01. ^*^p < 0.05.

We examined whether the mobility of MCF-7 cells was increased by the downregulation of SDC1 by shRNAs or miR-122-5p. We suppressed SDC1 expression by transfection of shSDC1, the gene-specific shRNA to suppress SDC1 expression. We also transfected a SDC1-expression construct to overexpress SDC1 protein in MCF-7 cells (Figure 4A). As shown in Figure 4B and Supplementary Figure 2A, the suppression of SDC1 expression by miR-122-5p or shSDC1 increased cell mobility, and the overexpression of SDC1 reduced cell mobility. This was consistent that the migratory ability of breast cells was inversely correlated with the levels of the SDC1 protein [16], which were regulated by miR-122-5p. Downregulation of miR-122-5p (Supplementary Figure 1A and 1B) promoted upregulation of SDC1 in the clinical samples (Figure 1A), admission or upregulation of miR-122-5p might suppress the malignancy of breast cells in term of cell proliferation, but potentially enhanced cell mobility.

### Presence of miR-122-5p in hepatoma-derived exosome regulated breast cancer cell mobility

The miR-122-5p is specially enriched in liver tissue [23], that would be specific source to release miR-122-5p. Currently, it was known that intracellular miRNAs could be released as circulating miRNAs under specific circumstance through the machinery of exosomes [24, 25]. For instance, miR-122-5p was recognized as a specific marker of liver injury, and it was found in biofluid samples [25–27]. We had demonstrated that miR-122-5p suppressed SDC1 expression and enhanced the mobility of MCF-7 breast cancer cells, we examined whether liver-derived exosomes containing miR-122-5p would affect the mobility of breast cancer cells.

**Figure 5:**
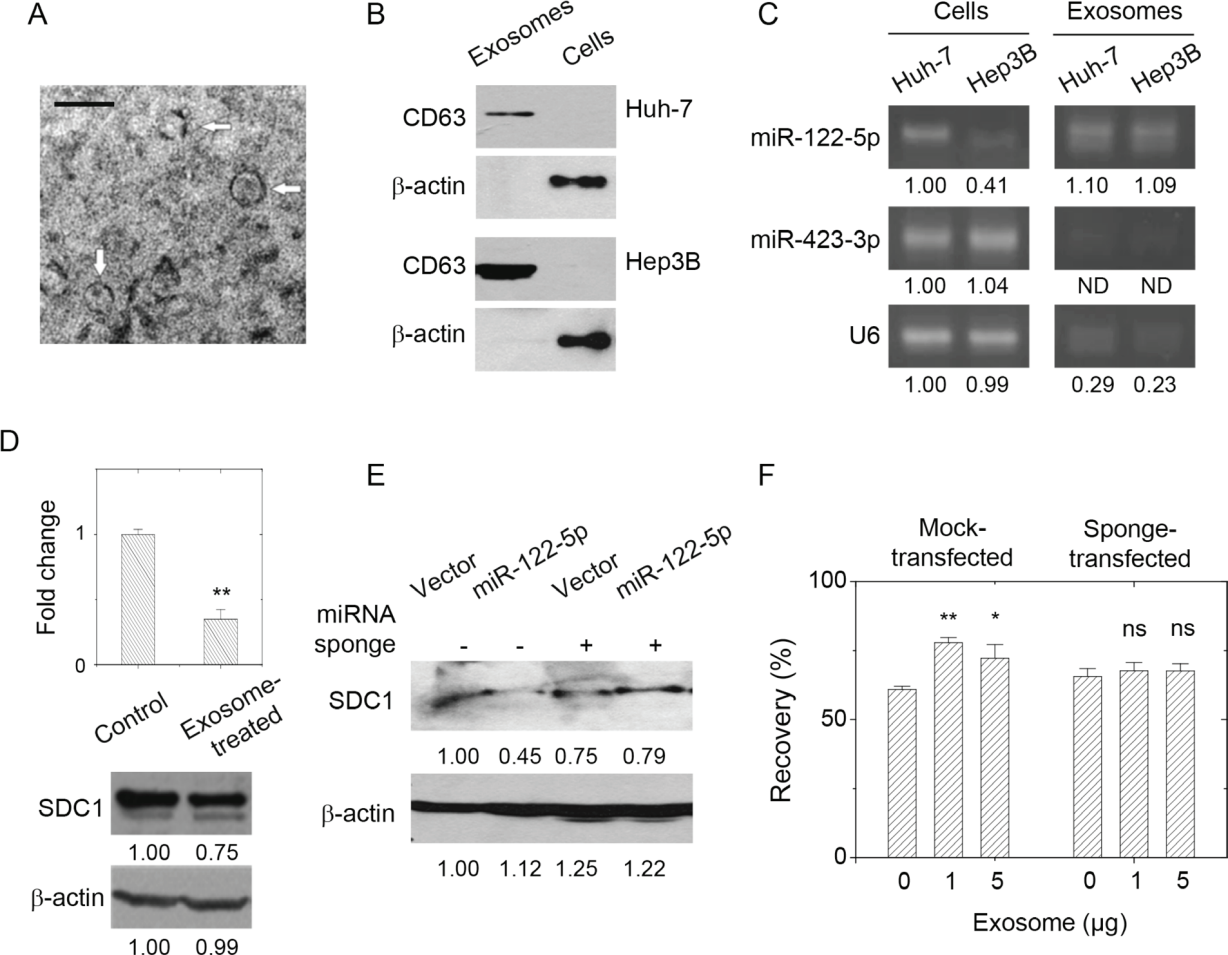
Presence of miR-122-5p in hepatoma-derived exosome affected breast cancer cell mobility through SDC1 down-regulation (**A**) Characterization of hepatoma cell-derived exosomes by transmission microscopy. Bar = 100 nm. (**B**) Characterization of hepatoma cell-derived exosomes by the presence in CD63 by western blot. (**C**) The level of miR-122-5p in hepatoma cells and hepatoma cell-derived exosomes as analyzed by PCR. It showed the enrichment of miR-122-5p in the exosomes, but not for miR-423-3p (control) or U6 small RNA. (**D**) Treatment of breast cancer cells with hepatoma cell-derived exosomes (5 μg) gave decreased SDC1 mRNA and protein expression. (**E**) Transfection of miR-122-5p-specific sponge into MCF-7 cells rescued the suppression of SDC1 downregulation by miR-122-5p (the third and fourth lanes) comparing with those transfected with mock sponge (the first and second lanes). (**F**) Treatment of MCF-7 cells with hepatoma cell-derived exosomes increased cell mobility as observed by increased recovery in wound healing assay (in mock-transfected cells). The presence of miR-122-5p-specific sponge abolished the effect of exosome treatments. Data were mean ± S.E. ^**^p < 0.01, ^*^p < 0.05. *ns*: not significant.

Exosomes secreted from hepatoma cells were characterized with transmission electron microscopy and western blot. As shown in Figure 5A, positive-negative contrast staining showed exosomes that were approximately 30 nm in diameter. The sizes of exosomes typically ranged from 30 to 100 nm depending on the cell source. We further characterized the presence of exosome-specific marker protein CD63 [28] by western blot. As shown in Figure 5B, CD63 was present in exosomes derived from hepatoma Huh-7 or Hep3B cells. In contrast, intracellular levels of CD63 were hardly seen in Huh-7 and Hep3B cells. The levels of miR-122-5p in the hepatoma-derived exosomes were characterized by PCR. As shown in Figure 5C, miR-122-5p was present inside both the Huh-7 and Hep3B cells, and in both cell-derived exosomes. The relative contents of miR-122-5p in Hep3B cell-derived exosomes were more than that inside Hep3B cells. As to miR-423-3p, it showed high level in Huh-7 or Hep3B cells, but was absent in cell-derived exosomes. The small RNA loading control U6-RNA was present in hepatoma cells but it was also rare in the cell-derived exosomes. This suggested the enrichment of miR-122-5p in exosomes secreted from both Huh-7 and Hep3B hepatoma cells. There would exist one unique mechanism to enrich miR-122-5p.

Interestingly, the treatment of MCF-7 cells with hepatoma-derived exosomes reduced both the levels of SDC1 mRNA and protein expression (Figure 5D). The miR-122-5p expressing vector slightly reduce SDC1 mRNA of MCF7 cells (data not shown) but reduced SDC1 protein expression (Figure 3A), but treatment of miR-122-containing exosome significantly reduced SDC1 mRNA (Figure 5D). We suspected other exosomal components contributed to this effect. To examine whether the effects of exosome treatment correlated with the function of miR-122-5p, we generated a construct containing the complementary sequence of miR-122-5p, which acted as a miRNA-specific inhibitor or so-called miRNA sponge [29]. As shown in Figure 5E, miR-122-5p sponge interfered with the ability of miR-122-5p in downregulation of SDC1 expression. MCF-7 breast cancer cells were treated with hepatoma-derived exosomes, and then wound healing assays were performed. As shown in Figure 5F and Supplementary Figure 2B, the treatment of MCF-7 cells with exosomes enhanced cell mobility. However, the mobility of sponge-transfected MCF-7 cells was not affected by exosome pretreatment. Our results indicated that the liver-derived exosomes increased the mobility of breast cancer MCF-7 cells though SDC1 downregulation mediated by exosomal miR-122-5p. We had found that the exosomal level of miR-122-5p was increased upon hepatoma cell damage treated by apoptotic agent (Supplementary Figure 1F). We suggested that liver injury might be the risk factor associated with metastasis of breast cancer cells. The mechanism of increased cell mobility would be mediated by SDC1 downregulation contributed by exosomal miR-122-5p.

## DISCUSSION

SDC1 in breast carcinomas was correlated with poorer prognosis and an aggressive phenotype [7]. Membrane-bound SDC1 promoted cell proliferation but inhibited cell invasiveness; while soluble SDC1 that shed from cell membrane played the opposite role [16]. It had been published that SDC1 could be regulated by miR-10b in breast cancer [21], miR-143 in melanoma [30], miR-126 and -149 in prostate cancer [31], and miR-145 in urothelial carcinoma [32]. Although miR-10b-5p was downregulated in breast malignancy as analyzed in GEO data (Figure 2A), RNA22 analysis did not give significant binding (−25 kcal/mol). This might be due to less continuous pairing in the duplex structure [21].

Although tumor malignancy correlated with the potential of tumor development and metastasis, we suggested the potential of tumorigenesis might not directly coupled with potential of tumor metastasis. Tumor development in the view of cell proliferation depends on extracellular stimuli though cell surface receptor or proteoglycans. Upregulation of SDC1 would promote the progression and transformation of breast cancer (Figure 1A). Our unpublished data showed miR-122-5p suppressed breast cancer cell proliferation as similar to the previous literature [33]. However, tumor cell mobility is more complicated and determined by the context of cell-matrix interaction, matrix degradation, as well the rearrangement of intracellular cytoskeleton. Our observation at downregulation of SDC1 expression did enhance cell mobility, which was consistent with previous observation [21].

According to the GEO data (Figure 1A), progression of breast cancer was correlated with upregulation of SDC1 expression. Recently, one GEO dataset (GSE86995) provide the information on the association of breast cancer malignancy with microRNA expressions. The miR-122-5p was in higher level in distant metastases than in primary breast tumors either as DCIS or primary IDC. This suggested the acquisition of high mobility through epithelial-mesenchymal transition from primary cancer cells into metastatic breast cancer cells [21]. The breast cell mobility was increased upon SDC1 downregulation by admission of miR-122-5p (Figure 4B) or liver-derived exosome treatment (Figure 5F). This proposed one possible mechanism to explain the metastasis of breast cancer cells and the association with acute liver failure [9, 10]. Recent paper indicated that higher level of circulating miR-122 associated with metastasis in breast cancer patients, which was explained by suppression of glucose uptake by niche cells but increase of glucose uptake by breast cancer cells using cancer-cell-secreted miR-122 [34]. However, it still lacks direct evidences to support our mechanism that liver-derived exosomes released at chronic normal condition or under acute liver damage might promote distant metastasis of breast cancer from primary tumors. Nevertheless, it is worthy to evaluate and develop anti-miR-122-5p strategy after initial therapy to prevent distant metastasis. In addition, specific evaluation at other exosomal components, including other miRNAs, mRNA, and proteins, that might affect and alter the cellular activities of target cells.

## MATERIALS AND METHODS

### Cell culture

Human hepatoma Huh-7, Hep3B cells, human embryonic kidney Hek293 cells, breast carcinoma MCF-7, and MDA-MB-231 cells were all purchased from Bioresource Collection and Research Center (BCRC; Hsinchu, Taiwan) with authentication. They were maintained in DMEM medium supplemented with 10% (v/v) fetal bovine serum (FBS) at 37° C under 5% CO_2_ and 100 % humidity. Plasmid transfection was done using transit-LT1 transfection reagent (Mirus Bio LLC., Medison, WI, USA) according to the manufacturer's instruction. Transfected cells were selected and enriched with culture medium containing G418, hygromycin B, or puromycin. For the treatment of exosome at MCF-7 cells, 2 × 10^5^ cells were plated overnight and incubated at serum-free medium for 4 hr. Exosome solution with desired protein equivalent was added and pipetted gently into the medium and kept at incubator for additional 24 hr. The cells were then harvested for PCR, or western blot.

### Exosome preparation

The exosome-producing medium was prepared to remove residual exosomes from FBS as referred [35] with modification. Generally, 50% (v/v) FBS in DMEM medium was centrifuged at 2,000 × g for 10 min, and then centrifuged at 100,000 × g (Beckman Optima L90-K with 90Ti rotor; Beckman Coulter Taiwan Inc., Taipei, Taiwan) for 16 hr at 4° C. The supernatant were collected and diluted into 10% (v/v) FBS by serum-free DMEM, and were sterile through 0.22 μm filter. For production of hepatoma-derived exosomes, 1 × 10^6^ Hep3B or Huh7 cells were plated in culture medium overnight, and were replaced into exosome-producing medium for successive culture for 2 days. Exosome-containing medium (100 mL) were collected and exosomes were isolated by Total Exosome Isolation kit (Life Technologies, Grand Island, NY, USA) according to suggested protocol. The pellets containing secreted exosomes were further washed by DEPC-treated PBS, centrifuged at 100,000 × g for 60 min (Beckman Optima MAX-E with TLA-120.2 rotor), and repeated twice to remove residual serum protein. The protein content in the exosome solution was determined by protein assay reagent (Thermo Fisher Scientific Inc., Pittsburgh, PA, USA).

### PCR

The levels of mRNA in cultured cells were analyzed by PCR. The total RNA was extracted using Trizol reagent (Life Technologies, Grand Island, NY, USA). The cDNAs were synthesized by MMLV HP reverse transcriptase (Epicentre, Madison, WI, USA) according to vendor's protocol. PCR reaction was done using McTaq DNA polymerase (Won-Won Biotechnology, Co. Ltd.., Taishan, New Taipei City, Taiwan), freshly prepared cDNA pools and specific primers. PCR reactions were carried out using gene-specific primers: human SDC1; 5ʹ-gctctggggatgactctgac-3ʹ and 5ʹ-gtattctcccccgaggtttc-3ʹ; human GAPDH; 5ʹ-gagtcaacggatttggtcgt-3ʹ and 5ʹ-gatctcgctcctggaagatg-3ʹ. Quantitative real-time PCR were performed using VeriQuest Fast SYBR green qPCR reagent (Affymetrix Inc., Santa Clara, CA, USA) in a StepOne Plus real-time PCR system (Thermo Fisher Scientific Inc., Pittsburgh, PA, USA). The 2^–ΔΔCT^ method was used to determine the relative gene expression using GAPDH as control. For PCR analysis of mature miRNA, the small RNAs were purified by miRNA isolation kit (Geneaid biotech Ltd., Shijr, NewTaipei City, Taiwan). The specific RT primer for reverse transcription of small RNAs into cDNA was listed in Supplementary Table 1. For PCR assay, the DNA segment corresponding to mature miRNA and one universal reverse primer (see Supplementary Table 1) were used as forward primer and reverse primer, respectively.

### Plasmid constructions and preparation

The recombinant DNA experiments were practiced under the National Institutes of Health Guidelines and approved by Fu Jen Catholic University Biosafety committee with approval number B9712. The shRNA constructs for SDC1 silencing were purchased from National RNAi Core Facility located at the Institute of Molecular Biology / Genomic Research Center, Academia Sinica, NanKang, Taiwan. The construction for miR-122 overexpression plasmid was done by the following procedure. PCR cloning was done by Unipol enzyme mixture (Ampliqon, Skovlunde, Denmark). The primers used for cloning of pri-miR-122 sequence are listed in Supplementary Table 1. The PCR product was then digested by *Bgl*II and *Hind*III (Thermo Fisher Scientific Inc., Pittsburgh, PA, USA) and ligated with the pEGFP-N1 plasmid (kindly gifted from Dr. Burton Yang, Sunnybrook Health Science Center, ON, Canada) digested with the same enzymes. Cloning of *SDC1* coding sequence or 3′UTR sequence was done by PCR using UniPol enzyme mixture, cDNA pools from MCF-7 cells, and specific primers (see Supplementary Table 1). The PCR product was digested by *Spe*I and *Hind*III, and then ligated into the pMIR-REPORT™ luciferase vector (Thermo Fisher Scientific Inc., Pittsburgh, PA, USA) predigested with the same enzymes. The miR-122-5p sponge and mock sponge (as control) were designed to suppress miR-122-5p inhibitory activity accordingly [29], and were constructed using specific primers (Supplementary Table 1). The mock plasmid was designed to act complementary against CXCR4 sequence as referred [29]. The PCR product was digested by *Hind*III and *Xho*I, and then ligated into the pCDNA3.1 vector predigested with the same enzymes. The ligated plasmids were then transformed into HIT^TM^-DH5α cells (Real Biotech Corporation, NewTaipei City, Taiwan) and single bacterial colonies were selected. They were amplified, isolated from bacteria, and sequence-characterized to confirm the fidelity of clones.

### GEO data analysis

SDC1 expression data from GEO datasets were extracted using interactive webtool GEO2R; https://www.ncbi.nlm.nih.gov/geo/geo2r/. SDC1 expression level increased in cancerous tissues (GDS3853), but not significantly changed with tumor subtypes, grades, or stages (GDS4056 and GDS4057). The miRNA expression data in GSE7842 were grouped into normal tissues and tumor tissues regardless of tumor subtypes, grade or stages. The data for each miRNA expression were averaged, and relative changes were calculated using the formula: relative change (%) = [(expression in tumor tissues - expression in normal tissues)/(expression in normal tissues)] × 100.

### Multiplex PCR

For *in vitro* interactions of miRNAs with 3′UTR of SDC1, one multiplex PCR approach [20] was adapted. The pcDNA3.1 plasmid containing 3′UTR of SDC1 was used as template. Two different forward primers were used in two independent PCR reactions. Forward primer 1 (fp1, Supplementary Table 1) targeted at starting of 3′UTR of SDC1 gene; while forward primer 2 (fp2, Supplementary Table 1) located at 200-bp downstream of fp1-targeting site. Nucleotide correspond to mature miRNA sequence was used as reverse primer in multiplex PCR. The PCR mixture contained reaction buffer, plasmid template, 10 μM dNTPs, 1 μM primer, and 1 U DNA polymerase. The parameters for the PCR reaction were: one cycle at 94° C for 10 min; 35 cycles at 94° C for 2 min, 42° C for 2 min, 72° C for 2 min; and a final elongation step at 72° C for 10 min. The PCR products were then visualized with a 1.5% agarose gel. The histogram was generated and analyzed by Image J [36].

### Western blot

For western blotting analysis, cells were washed, disrupted by lysis buffer of 10 mM Tris-HCl, 5 mM EDTA, pH 8.0, 1 (w/v) % TritonX-100, and protease inhibitors (Sigma-Aldrich Inc., Shanghai, China) and kept on ice for 30 min. The lysate was then centrifuged at maximum speed using a desktop centrifuge at 4° C for 10 min. Protein concentrations were quantified by protein assay kit (Bio-Rad Laboratories Inc., Hercules, CA, USA).

For western blot characterization of SDC1 protein, 50 μg cell lysate was pretreated with 0.83 mIU heparinase I, 0.83 mIU heparinase II, 0.83 mIU heparinase III, and 0.83 mIU chondroitinase in 200 ul reaction buffer (20 mM Tris-HCl, pH 7.5, 4 mM CaCl_2_, and 0.1 % (w/v) BSA) at 37° C for 16 hr. The proteins were precipitated by trichloroacetic acid and solubilized by solubilizing solution (10 mM Tris-HCl, pH8.0, 5 mM EDTA, and 2 M urea) then subjected to SDS-PAGE, and transferred onto a PVDF membrane. Standard western blot procedure was performed using primary antibody (B-A38; 1:1000) for 2 hr at room temperature, and HRP-conjugated secondary antibody (1:2000) for 1 hr at room temperature. The blots were visualized by enhance chemiluminescent detection (EMD-Millipore, Inc., Billerica, MA, USA). The primary antibody against CD63 and β-actin were obtained from GeneTex Inc., Hsinchu, Taiwan.

### Wound healing assay

Wound healing assay was used to evaluate cell mobility by filling the gap between confluent MCF-7 cells. The gaps with ~500 um width were generated using silicon culture insert (Ibidi GmbH, Planegg, Germany) according to manufacturer's instruction. In general, 7 × 10^4^ cells were plated at each well of insert. The culture inserts were removed carefully. Cell layers were carefully washed by PBS, and supplied with culture medium for gap closure. For exosome treatments, exosomes with desired protein equivalence were pretreated upon cell seeding and incubated overnight before insert removal. The gap images at the same locations were recorded under microscopy at different time intervals. The percent recovery of gap was calculated by the formula: (the empty area at specific time interval / the area right after the insert removal) × 100%.

### *In vitro* luciferase activity assay

Luciferase activity assays were performed using pMIR-REPORT™ miRNA expression reporter vector system (Thermo Fisher Scientific Inc., Pittsburgh, PA, USA). The vectors containing luciferase gene with or without 3′UTR of *SDC1* were transfected into cells and compared the luciferase activities in the presence of miRNA-122-5p expression. In general, Hek293 cells were seeded (4 × 10^4^ cells per well) into 24-well plates till 70~80% confluence. Totally 1 μg DNAs containing luciferase-UTR construct, β-Gal vector, and miRNA-expression vector with the ratio of 1:1:10 were transfected using Turbofect transfection reagent (Thermo Fisher Scientific Inc., Pittsburgh, PA, USA). Cells were then harvested using trypsin/EDTA, and the β-Gal activity and luciferase activity were assayed using luciferase assay system (Promega corporation, Madison, WI, USA). Experiments were repeated four times and luciferase activities were normalized against β-Gal activity.

### Transmission electron microscopy analysis of exosomes

The preparation of grids containing hepatoma-derived exosomes was performed accordingly [35]. In general, 5 μL of paraformaldehyde-fixed exosome solution was coated onto a formvar-carbon coated EM grid (Electron Microscopy Sciences, Hatfield, PA, USA). The contrast staining by uranyl-oxalate solution and successive methyl cellulose-uranyl acetate solution was conducted on ice. The imaging of hepatoma-derived exosomes was taken by JEOL JEM-1400 transmission electron microscopy (JEOL USA Inc., Peabody, MA, USA) at 80 kV.

### Statistical analysis

The statistical analysis of qPCR data or GEO data were done by Origin7.0 software (OriginLab Corporation, USA) using unpaired/one-tailed two sample *t*-test. The *p*-value of < 0.05 was considered as statistically significant. The *p*-value of < 0.01 was considered as extremely significant.

## SUPPLEMENTARY MATERIALS FIGURES AND TABLES



## Abbreviations

SDC1: Syndecan-1
miRNA: MicroRNAs
3ʹUTR: 3ʹ-untranslated region
GEO: Gene Expression Omnibus
FBS: fetal bovine serum
DCIS: ductal carcinoma *in situ*
IDC: invasive ductal carcinoma
