# Supplementary material for: Mining of potential microRNAs with clinical correlation - regulation of syndecan-1 expression by miR-122-5p altered mobility of breast cancer cells and possible correlation with liver injury
Source: Oncotarget. 2018 Jun 15;9(46):28165–75. doi: 10.18632/oncotarget.25589 (PMC6021336; doi:10.18632/oncotarget.25589)
Supplement: Supplementary file 1 [file oncotarget-09-28165-s001.pdf]

Mining of potential microRNAs with clinical correlation - regulation of syndecan-1 expression by miR-122-5p altered mobility of breast cancer cells and possible correlation with liver injury

SUPPLEMENTARY MATERIALS

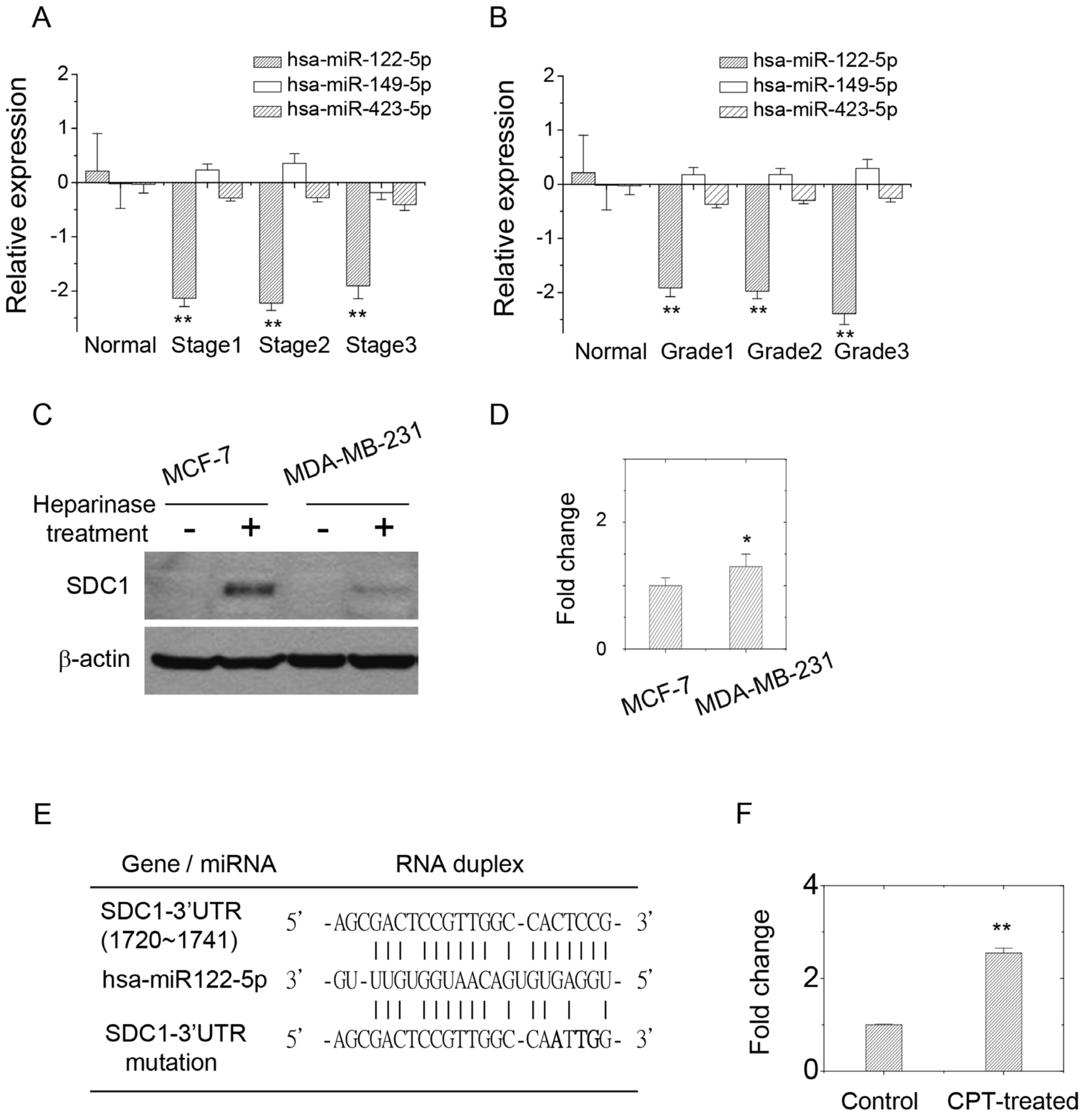

Supplementary Figure 1: Extracted data in GSE7842 for expression of miR-122-5p under (**A**) different patient stages and (**B**) tumor grades. Data were mean ± S.E. $^{**}p < 0.01$. (**C**) SDC1 protein expression in MCF-7 cells ($ER^{+}PR^{+}$; luminal type) was higher than MDA-MB-231 cells (triple negative; basal type). (**D**) The miR-122-5p expression level was relatively higher in MDA-MB-231 cell than in MCF-7 cells. Data were mean ± S.E. $^{**}p < 0.05$. (**E**) RNA duplex for hsa-miR-122-5p pairing with SDC1-3′UTR or SDC1-3′UTR mutated at seed region. (**F**) The miR-122-5p expression level in hepatoma cells was increased upon treatment of chemoagent camptothecin (CPT; in 10 μM) as quantified by qPCR. Data were mean ± S.E. $^{**}p < 0.05$.

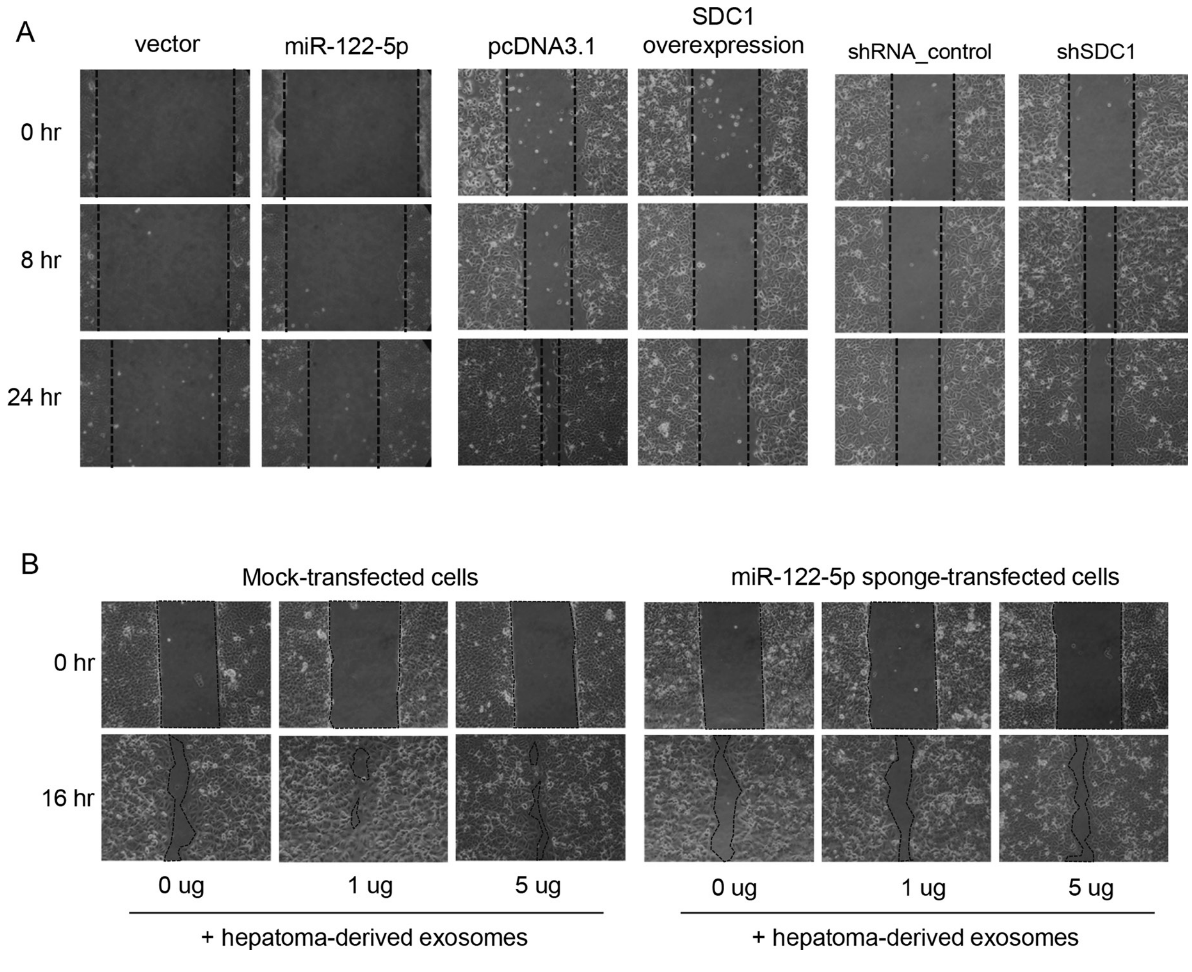

Supplementary Figure 2: (**A**) Effect of transfection of miRNA-122-5p, SDC1 construct, or shRNA against SDC1 on MCF7 cell mobility as observed by wound healing assay. (**B**) Effect of treatment of hepatoma-derived exosome at MCF7 cell mobility with or without miR-122-5p-specific inhibitor (miRNA sponge) as observed by wound healing assay.

Supplementary Table 1: Primers used for construction of miR-122-5p overexpression plasmid, SDC1 overexpression plasmid, SDC1-3′UTR, SDC1-3′UTR mutation, miR-122-5p sponge, and PCR analysis of miR-122 expression

Primer	Sequence
Forward primer for miR-122-5p cloning	5′-cctgtgatagatctccttagcagagctgtggagtgtgacaatggtgtttgtgtctaaactatcaaacgcc-3′
Reverse primer for miR-122-5p cloning	5′-cctgtgataagcttgcctagcagtagctatttagtgtgataatggcgtttgatagtttagacacaaac-3′
Forward primer for SDC1 CDS cloning	5′-cctgataagcttatgaggcgcgcggcgctctgg-3′
Reverse primer for SDC1 CDS cloning	5′-cctgatctcgagtcaggcatagaattcctcctgtttggtgg-3′
Forward primer for SDC1 3′UTR cloning	5′-cctgatactagtcgcgggagccatgcgcc-3′
Reverse primer for SDC1 3′UTR cloning	5′-cctgataagctttttgcccgaaatgacaagtaccg-3′
Forward primer for SDC1 3′UTRmut cloning	5′-cctgatgaattcgaagacccctggcagctcccgagc-3′
Reverse primer for SDC1 3′UTRmut cloning	5′-cctgatctcgagaatggagggaagggacaaagaagatagaga-3′
Forward primer 1 (fp1) in multiplex PCR	5′- aattctatgcctgacgcgggagccatg -3′
Forward primer 2 (fp2) in multiplex PCR	5′- aggggtttctcgcataggac -3′
RT primer for miRNA assay	5′- gcgagcacagaattaatacgactcactataggttttttttttttvn -3′
Forward primer for miR-122-5p PCR	5′- tggagtgtgacaatggtgtttg -3′
Forward primer for miR-423-3p PCR	5′- tgaggggcagagagcgagacttt -3′
Forward primer for U6 RNA PCR	5′- gtgctcgcttcggcagcaca -3′
Universal reverse primer for PCR	5′-aattctaaattatctaatataggagatgaattcttatgatattac-3′
Forward primer to clone miR sponge	5′-cctgataagcttcaaacaccaacagacactccaccggcaaacaccaacagacactccaccggcaaacaccaacag acactccacc-3′
Reverse primer to clone miR sponge	5′-cctgatctcgagccggtggagtgtctgttggtgtttgccggtggagtgtctgttggtgtttgccggtggagtgtctgttg gtgtt-3′
Forward primer to clone mock sponge	5′-cctgataagcttaagttttcagaaagctaacaccggaagttttcagaaagctaacaccggaagttttcagaaagctaac acc-3′
Reverse primer to clone mock sponge	5′-cctgatctcgagccggtgttagctttctgaaaacttccggtgttagctttctgaaaacttccggtgttagctttctgaaa ac-3′
